# Evaluation of Different Commercial Sealing Hemostatic Patches for Their Selection as Reservoirs for Localized Intraperitoneal Chemotherapy

**DOI:** 10.1021/acsptsci.4c00608

**Published:** 2025-01-08

**Authors:** M. Teresa Perelló-Trias, Ana Rodríguez-Fernández, Antonio Jose Serrano-Muñoz, Juan J. Segura-Sampedro, Pedro Tauler, Joana M. Ramis, Marta Monjo

**Affiliations:** † Cell Therapy and Tissue Engineering Group (TERCIT), Research Institute on Health Sciences (IUNICS), University of the Balearic Islands (UIB), 07122 Palma, Mallorca, Spain; ‡ Health Research Institute of the Balearic Islands (IdISBa), 07010 Palma, Mallorca, Spain; § Department of Fundamental Biology and Health Sciences, University of the Balearic Islands (UIB), 07122 Palma, Mallorca, Spain; ∥ General & Digestive Surgery Service, Hospital Universitario la Paz, 28046 Madrid, Spain; ⊥ Faculty of Medicine, University of the Balearic Islands (UIB), 07122 Palma, Mallorca, Spain; # Research Group on Evidence, Lifestyles and Health, Research Institute of Health Sciences (IUNICS), University of the Balearic Islands (UIB), 07122 Palma, Mallorca, Spain

**Keywords:** hemostatic patch, drug delivery system, hydrogel, hyaluronic acid, localized intraperitoneal chemotherapy

## Abstract

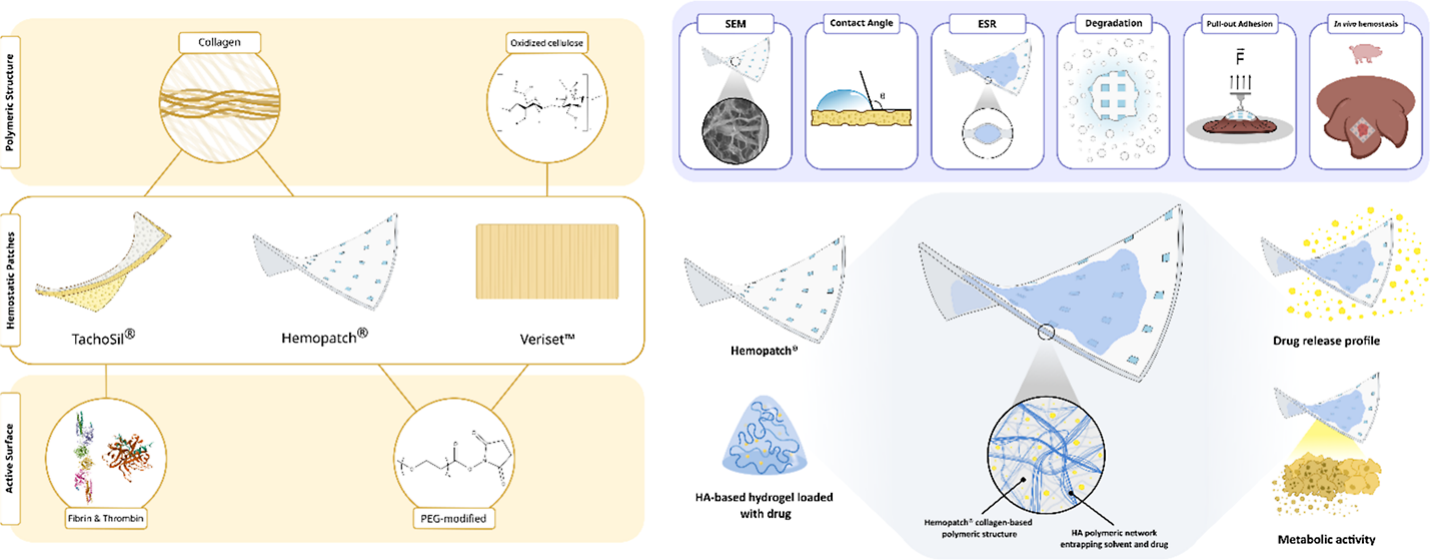

Peritoneal carcinomatosis (PC) is typically treated by cytoreductive surgery (CRS) and subsequent chemotherapy. Sealing hemostatic patches (HP) are often used during these surgeries to prevent complications such as uncontrolled bleeding. These HP are made of biomaterials like oxidized cellulose or collagen with a binding agent, and beyond their usual function, they could also be used as drug delivery systems (DDS) for localized intraperitoneal chemotherapy in the tissue attached. Our first aim was to characterize and compare three different commercial HP (TachoSil®, Hemopatch®, and Veriset^TM^). Hemopatch® emerged as the most suitable candidate due to its combination of properties, including slow degradation, high hydrophilicity, excellent biological fluid absorption capacity, and moderate adhesive capacity alongside hemostasis. Utilizing Hemopatch® as a scaffold, we developed a new device incorporating a hyaluronic acid hydrogel loaded with cisplatin or olaparib. This approach facilitated sustained drug release for over 6 days, maintaining the anticancer efficacy of these agents on OVCAR-3 cells. In conclusion, integrating a DDS into HP shows potential for precisely delivering chemotherapeutic agents to any residual microscopic disease in PC following CRS.

## 1

Cancer is currently one of the most prevalent diseases worldwide, and it stands as the second leading cause of death after cardiovascular diseases.^1,2^ Cancer originating from organs in the peritoneal cavity, such as the stomach, colon, liver, and ovary, can spread along the peritoneum, which is a condition known as peritoneal carcinomatosis (PC). This poses a significant challenge in managing the disease and is usually associated with decreased overall survival and poor prognosis.^3^ Current treatment for peritoneal malignancies involves cytoreductive surgery (CRS), followed by chemotherapy to remove microscopic residues.

**Figure 1:**
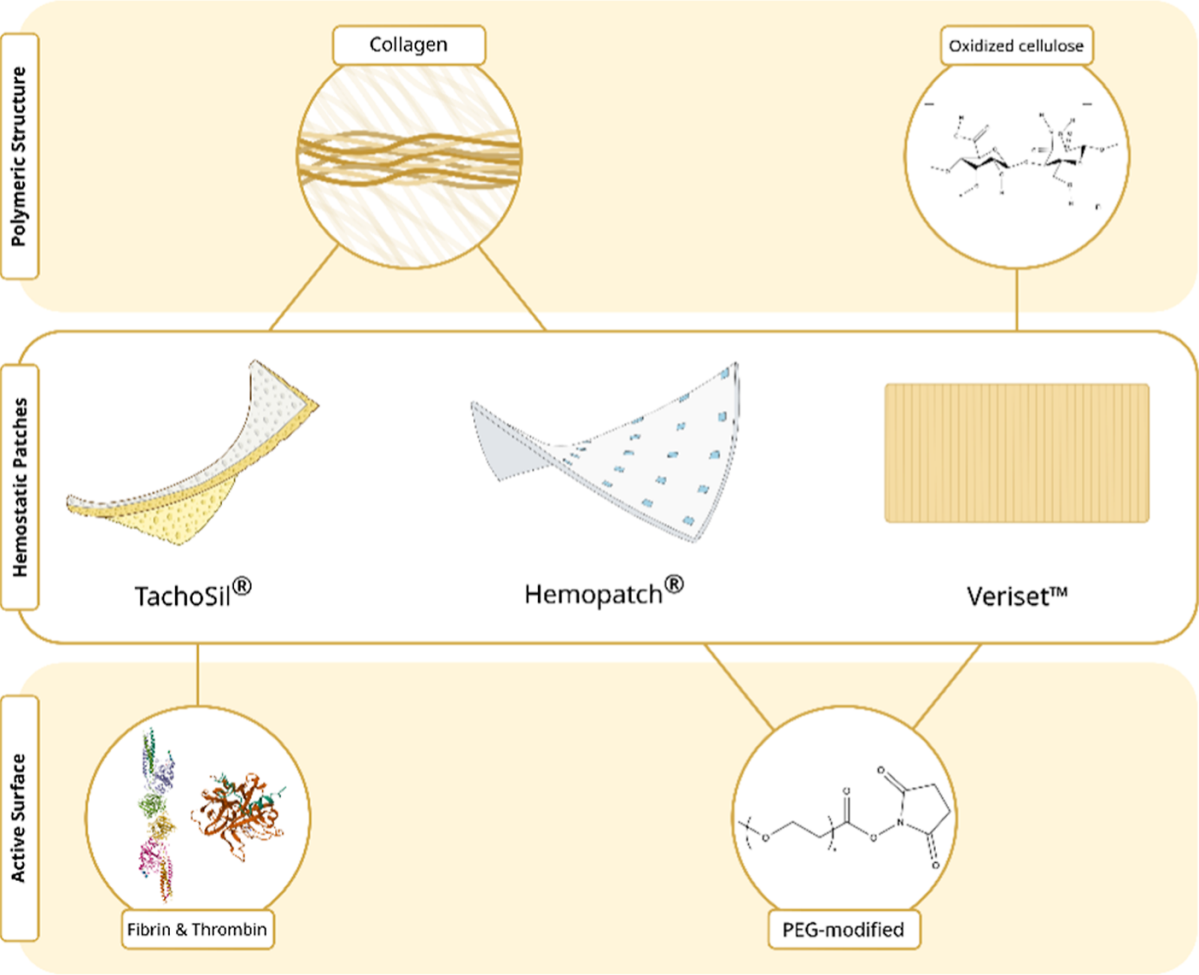
Graphical composition of three commercial hemostatic patches (TachoSil®, Hemopatch®, and Veriset^TM^) evaluated and characterized in the present study. PEG; polyethylene glycol. The fibrin image was obtained from the RCSB PDB (RCSB.org) of PDB ID 1FZC.^14^ The thrombin image was obtained from the RCSB PDB (RCSB.org) of PDB ID 1ETS.^15^

CRS aims to remove as much tumor tissue as possible. Still, it is also the first way to control the dissemination of metastatic cells in the peritoneal cavity. The remnant of cancer cells at the surgical resection margin appears to be a significant trigger for recurrences. Also, intraoperative bleeding due to tissue damage during tumor resection can be a critical factor in the dissemination of tumor cells into the blood and surrounding area, increasing the risk of new metastatic foci.^4,5^ Thus, adequate bleeding management during CRS is essential for intraoperative and postoperative complications as it can reduce drain placement and hospitalization time, morbidity, mortality, postsurgery costs, and metastasis events.^6−8^ Techniques such as electrocautery, compression, or ligatures are commonly used to achieve primary hemostasis.^9^ When these types of procedures are ineffective, surgeons may use other devices called local hemostatics, which differ in composition, mechanism of action, and formulation, such as liquid hemostatics, powders, fluids, etc.^10,11^ Among them, special mention should be made to hemostatic patches (HP),^12^ consisting of an absorbent sponge made of collagen or oxidized cellulose with an adhesive surface containing either fibrinogen and thrombin or a protein-reactive monomer, which allows tissue attachment and hemostasis.^13^ Among the different HP that are commercially available, TachoSil®, Hemopatch®, and Veriset^TM^ are widely used to control bleeding in surgery (Figure 1).

Intraperitoneal chemotherapy has emerged as a promising postoperative treatment for cancer patients by delivering chemotherapy directly into the peritoneal cavity. Cisplatin (CDDP), a platinum-based chemotherapeutic drug, is one of the most active cytotoxic agents and has been widely used in the treatment of PC by the intraperitoneal route.^16^ However, the application of CDDP in clinical practice is limited due to its severe dose-limiting toxicity and nonspecific activity.^17^

To address the limitations of conventional chemotherapeutic drugs, recent research has focused on advancing targeted chemotherapy to minimize toxicity to normal cells. One prominent approach is the use of poly(ADP-ribose) polymerase inhibitors (PARPi). Olaparib (OLA), the first PARPi, has been approved by the FDA for the oral treatment of advanced ovarian cancer.^18,19^ However, its clinical efficacy is currently far from ideal, mainly due to its reduced bioavailability after first-pass metabolism and low accumulation in tumor tissues.^20^

Different intraperitoneal chemotherapy administration strategies are being studied to provide effective drug concentration in the peritoneal cavity for an extended period and consequently improve the local control of peritoneal metastases after CRS.^21^ Thus, multiple drug delivery systems (DDS) are being developed to control the release of chemotherapeutics and overcome the drawbacks of conventional therapy, as recently reviewed.^22^

In the field of DDS, hydrogels are extensively used as three-dimensional hydrophilic polymer networks for drug encapsulation, offering increased drug solubility and permeability.^23^ However, their use for intraperitoneal chemotherapy must face some drawbacks, such as common initial burst release effects of small drugs and their dissemination within the peritoneal cavity.^21^ Hyaluronic acid (HA) is a natural polysaccharide that is well suited for DDS due to its chemically modifiable functional groups. These HA-derived polymers allow cross-linking into a hydrogel, enabling efficient drug encapsulation and prolonged release rates.^24^

The main objective of this study was to evaluate three different types of commercial HP to identify the most suitable one for use as a scaffold of a DDS as the porous structure of these HP would be ideal for incorporating an HA-based hydrogel loaded with chemotherapeutic agents. Upon selecting the most appropriate commercial patch, we aimed to integrate an HA-based hydrogel loaded with either CDDP or an OLA and to evaluate its release profile. Additionally, we assessed the impact of the released drugs on the viability of ovarian cancer, OVCAR-3 cells. Our goal was to develop a treatment option for localized intraperitoneal chemotherapy in PC by combining the patch’s adhesion and hemostatic properties with the controlled and sustained drug release of the hydrogel. The tissue adhesive is designed to remain adhered to the damaged tissue postcytoreduction, promoting hemostasis, while the hydrogel ensures local delivery of the therapy to eradicate microscopic tumor residues.

## 2 Results

### 2.1 Characterization of Commercial Sealing Hemostatic Tissue Patches

**Figure 2:**
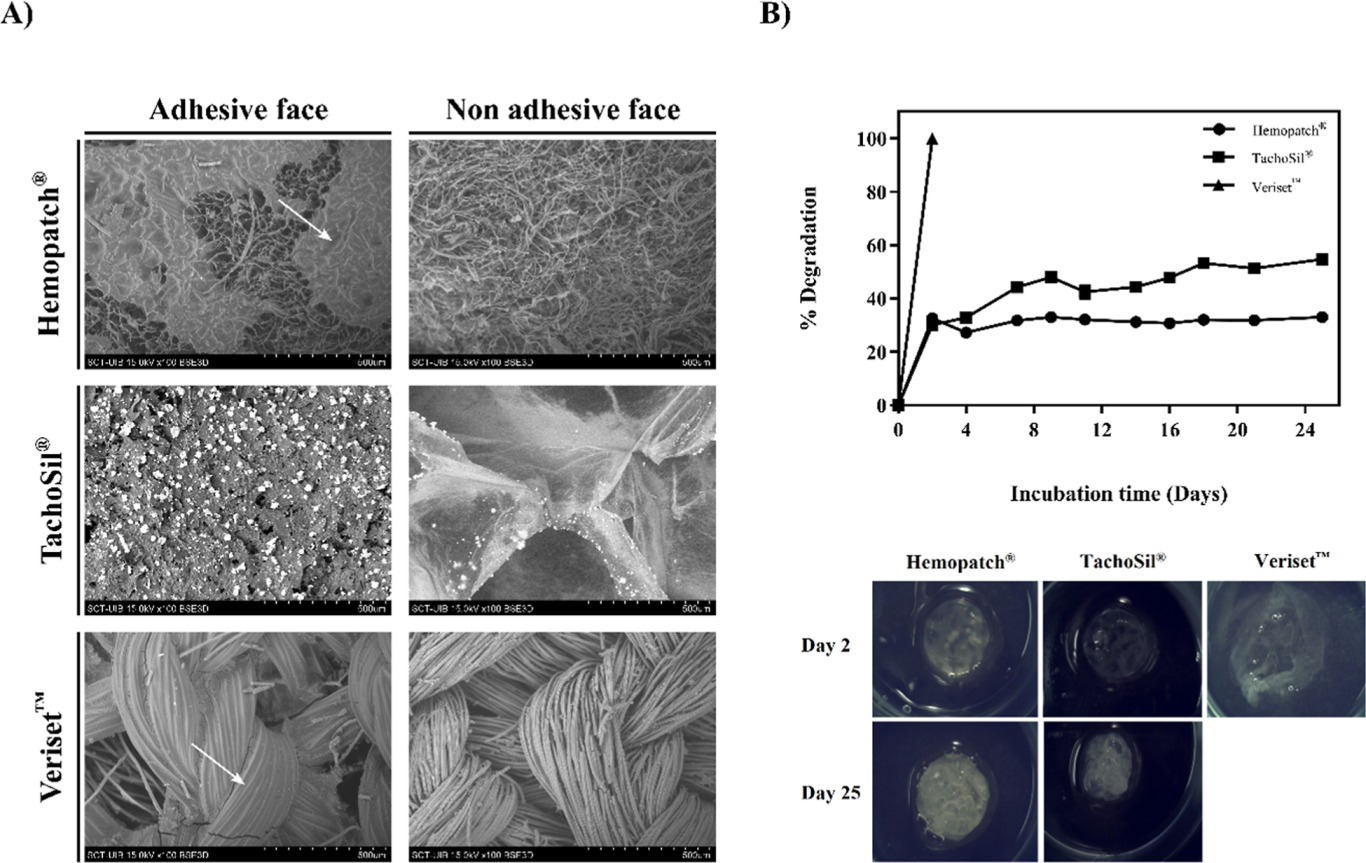
Characterization of commercial sealing hemostatic tissue patches. (A) Microstructure of the different patches. Images were taken by SEM at 15 kV, 40 Pa, and 100× magnification. The PEG layer is marked with white arrows. (B) Degradation data for three commercial HP after incubation with intraperitoneal liquid over time. Degradation profiles and images of different incubation times for the patches are shown. Values represent mean ± SEM.

Figure 2A shows images corresponding to the microstructure of the patches obtained by SEM, both on adhesive and nonadhesive sides, observing clear distinction between both surfaces, with adhesive surfaces featuring a coating of riboflavin (which imparts a yellow color), fibrinogen, and thrombin (TachoSil®), or polyethylene glycol (PEG) polymer (Veriset^TM^ and Hemopatch®). On the nonadhesive side, the structure of the sponge that acts as a support could be observed in greater detail. SEM images showed markedly porous structures for collagen patches with TachoSil® having much larger pore sizes than Hemopatch®. As for the cellulose patch Veriset^TM^, a braided system composed of many cellulose fibers was observed.

Next, the wettability of the different commercial patches was evaluated. The value for the contact angle was 88 ± 3° for TachoSil®, indicating that the patch surface was hydrophobic. On the contrary, Veriset^TM^ and Hemopatch® gave a high grade of hydrophilia and absorbed the liquid extremely fast, providing a contact angle of 0°.

The ability of water absorption was evaluated with ESR measurement (Table 1). After incubation, there was no significant increase in swelling data. The commercial patch Hemopatch® presented significantly higher values for ESR when compared with those of TachoSil® and Veriset^TM^. Veriset^TM^ showed a total degradation of the polymeric structure after 24 h of incubation, and thus, swelling results could not be obtained.

**Table 1:** ESR Data for Commercial HP at Different Incubation Times^a^

Incubation time (h)	Hemopatch®	TachoSil®	Veriset^TM^
0.5	0.083 ± 0.007	0.032 ± 0.002*	0.028 ± 0.008*
1	0.077 ± 0.003	0.038 ± 0.001*	0.030 ± 0.001*
3	0.096 ± 0.009	0.067 ± 0.003*	0.043 ± 0.006
24	0.079 ± 0.010	0.033 ± 0.002*	ND

a ESR data for incubation times of 0.5, 1, 3, and 24 h are shown (*n* = 3). The differences between groups were evaluated by the ANOVA test for parametric values and the Mann–Whitney test for non-parametric values. *p* < 0.05: * vs. Hemopatch®.

The degradation profiles of the different commercial patches were evaluated after incubation in ascitic fluid at 37 °C at various time points (Figure 2B). First, a significant difference can be observed between the degradation rates of collagen patches (TachoSil® and Hemopatch®) and the oxidized cellulose patch Veriset^TM^. Thus, Veriset^TM^ showed an almost complete degradation after 48 h, losing all its mechanical properties. Collagen patches exhibited a reduced rate of degradation over time, and Hemopatch® degraded the slowest. Curiously, during the incubation period, Hemopatch® formed an adhesive gel layer that seemed to increase its stability, delaying the degradation process.

**Figure 3:**
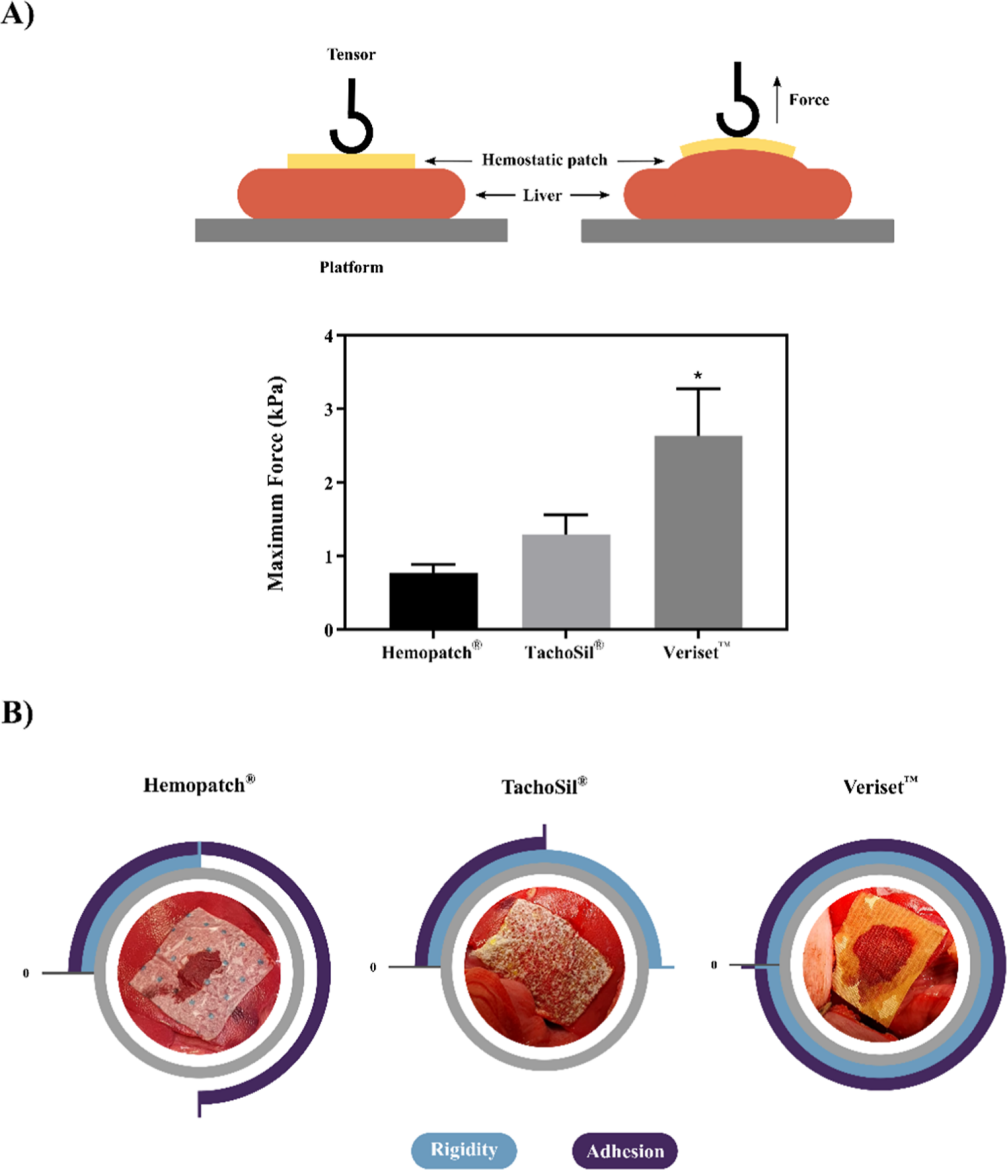
Ex and in vivo characterization of commercial sealing hemostatic tissue patches. (A) Pull-out adhesion measurement. The graphic shows the differences between the adhesion capacity of different commercial hemostatic patches when applying a tension force (*n* = 12). The values represent the mean ± SEM. The Kruskal–Wallis test evaluated the differences between groups. *p < 0.05: Hemopatch® vs Veriset^TM^. (B) Rigidity and adhesive capacity for commercial hemostatic patches in vivo after 3 min of compression (*n* = 3) and representative images after the application of the hemostatic patches. Data is presented in circular graphs according to a qualitative determination.

The adhesive capacity of the different patches was characterized by pull-out adhesion measurements (Figure 3A). A significant difference between the maximum force Veriset^TM^ could withstand before detaching from the tissue could be observed compared to Hemopatch®. There were no significant differences in adhesive capacity between TachoSil® and Hemopatch®.

Finally, the three patches presented good hemostasis in vivo, with 100% of the measurements indicating complete adhesion after 3 min of compression over the wound. Also, the rigidity of the various devices and the force needed to remove the patch after adhering it to the wound tissue were evaluated qualitatively by an experienced surgeon (Figure 3B). Veriset^TM^ presented higher adhesion values than the other patches with high rigidity, as observed in the ex vivo tests. Hemopatch® showed moderate adhesion and decreased rigidity, allowing easy product application. TachoSil® presented a moderate rigidity value but demonstrated low adhesion in wet tissues. Once removed, hemostasis was achieved in all cases.

### 2.2 In Vitro Study of CDDP and OLA Release from HAgel and HeHAgel and Effects on OVCAR-3 Cells

**Figure 4:**
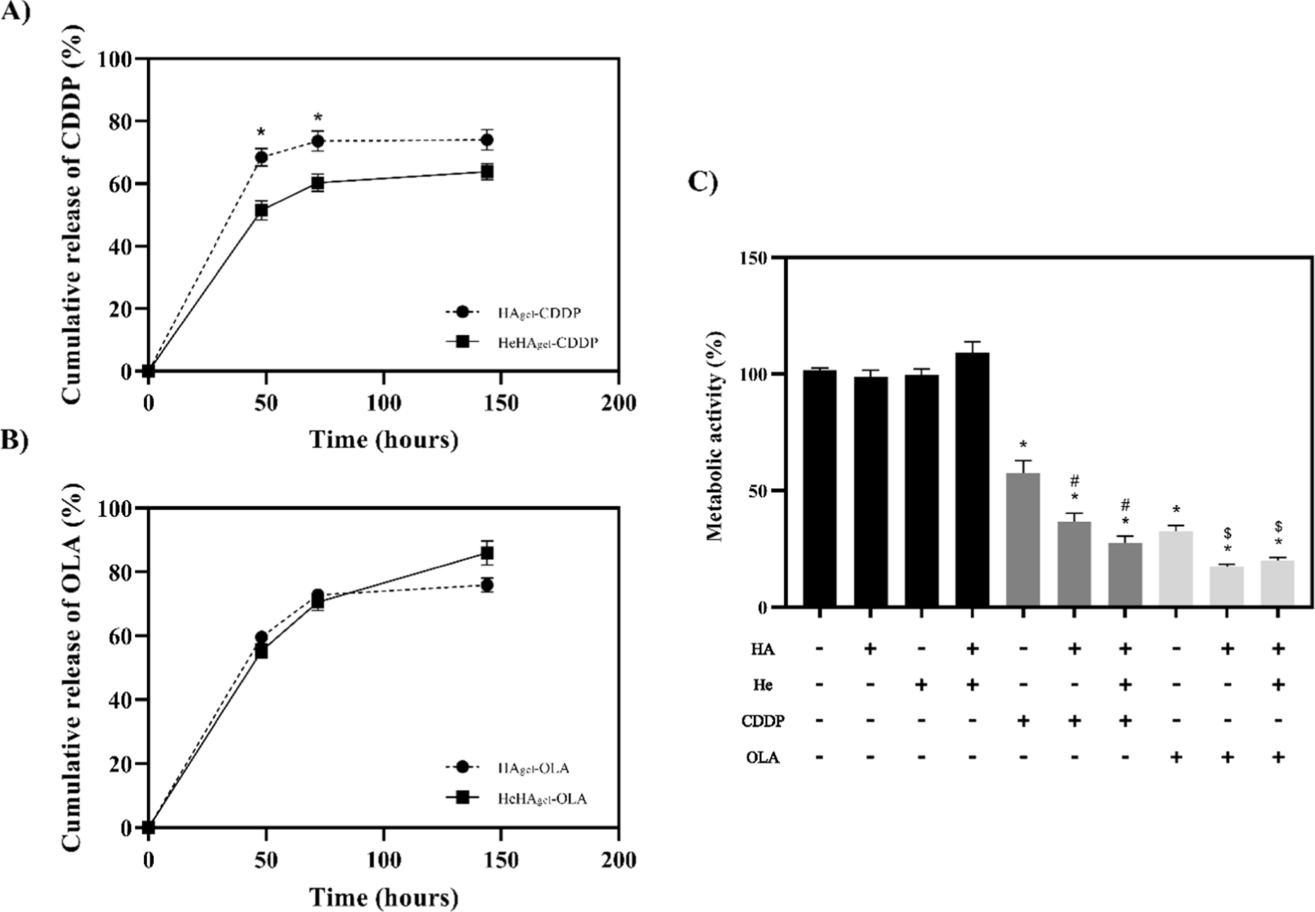
In vitro studies. (A) Release profile of CDDP from HAgel-CDDP and HeHAgel-CDDP at 48, 72, and 144h. Platinum content was measured by ICP–MS. (B) Release profile of OLA from HAgel-OLA and HeHAgel-OLA at 48, 72, and 144 h. OLA content was measured by HPLC with a diode array detector. Results were statistically compared by the ANOVA test. Three independent assays were performed (*n* = 9). Values represent the mean ± SEM *p < 0.05 vs HeHAgel-CDDP. (C) Metabolic activity of OVCAR-3 after 48 h of treatment. The data of the control group was set as 100%. Results were statistically compared by the Mann–Whitney test. Three independent assays were performed (*n* = 9). Values represent the mean ± SEM *p < 0.05 vs control, HA, He, and HeHA; #*p* < 0.05 vs IC_50_ CDDP and $ *p* < 0.05 vs IC_50_ OLA. HA: hyaluronic acid. He: Hemopatch®. CDDP: cisplatin. OLA: olaparib.

Due to the low degradation rate, super hydrophilicity, and high absorption capacity of biological fluids, together with a moderately good adhesive capacity and hemostasis, Hemopatch® was selected as a reservoir for DDS and used for further studies. Once we confirmed the correct drug encapsulation in the device due to the cross-linking and gelation properties of the two modified HA polymers (Figure 1S). HAgel containing a chemotherapeutic agent was applied to Hemopatch® for release and metabolic activity studies. Specifically, CDDP and OLA were chosen as model chemotherapeutic drugs to perform these determinations. All data was referred to a control group containing the maximum loading of CDDP or OLA in the hydrogel. The four devices gradually released CDDP (Figure 4A) or OLA (Figure 4B) over 6 days. Polymer gelation inside Hemopatch® caused a significantly slower CDDP release during the first 48 and 72 h compared to that with HAgel-CDDP alone, reducing the initial burst release. The same trend was observed when we evaluated the release at short time rates (Figure 2S). However, gelation inside Hemopatch® did not affect the OLA prolonged release profile. Next, the metabolic activity of the OVCAR-3 cells after 48 h of treatment was evaluated using a concentration of both chemotherapeutic agents corresponding to IC_50_ (Figure 4C). As expected, CDDP and OLA alone significantly reduced the metabolic activity of the OVCAR-3 cells, whereas no effect on cell viability was observed for HA and Hemopatch®, which were not loaded with drugs. For their part, CDDP and OLA loaded in HA and physically incorporated into Hemopatch® significantly reduced cell viability compared with their empty carriers. Moreover, the drug released from these devices inhibited cell viability more than the solution containing a concentration of CDDP or OLA equivalent to the IC_50_.

## 3 Discussion

HP are frequently employed during tumor resection in clinical practice. Apart from their primary function of stopping bleeding, these patches can act as scaffolds for localized treatment of residual neoplastic remnants at the microscopic level, making them a valuable tool in the surgical management of tumors. To effectively incorporate commercial patches into the treatment of PC, it is essential to carefully compare and select the one with the most suitable properties, including optimal microstructure and hydrophilicity for hydrogel loading, slow degradation rate to allow a progressive treatment release, suitable adhesion, and hemostatic properties, to treat the affected tissues locally. Thus, when combined with HA hydrogel loaded with chemotherapeutic agents such as CDDP or OLA, the chosen patch could be a reliable DDS, providing a sustained and localized release following CRS.

One of the main characteristics of these patches is their water absorption capacity since certain coagulation factors are released through the absorption of biological fluids. As we have observed, this absorption occurs rapidly, yet almost all of the commercial brands reached their maximum absorption capacity after half an hour. The Hemopatch® showed a higher absorption capacity, which could be related to the porous microstructure and the PEG polymer having a high affinity for water and retention capacity. On the other hand, the oxidized cellulose patch Veriset^TM^ lacks a porous structure. Still, the literature describes the capacity of this material to form a gel, which would allow the retention of liquids. Another important aspect is the differences found in the wettability. The only HP that allowed measuring the contact angle was TachoSil®, which was more hydrophobic, causing water to remain on the surface. This feature made TachoSil® not an optimal candidate for drug loading with HA hydrogels, but it still exhibited good hemostasis and sealing characteristics. Regarding Hemopatch® and Veriset^TM^, we observed zero wettability, presenting such a high affinity for water that contact angle measurements were not possible. This may be due to the presence of hydrophilic groups in the structures of the PEG polymers used as adhesives.

Although all three patches showed sufficient adhesion capacity, Veriset^TM^ showed the highest adhesive capacity after 3 min of pressure. This high adhesive capacity is linked to the presence of the adhesive polymer. However, the patch exhibits rapid degradation over time, which may be related to the quick degradation profile of oxidized cellulose. This challenges the use of the patch as a scaffold for incorporating the hydrogel with a chemotherapeutic drug. As described by other authors, oxidized cellulose is degraded following several pathways, such as decarboxylation and degradation by elimination facilitated by glucosidases.^25^ In studies conducted to determine the degradation mechanisms of oxidized cellulose materials, a higher degree of polymerization has been related to a lower presence of carboxyl groups, thus reducing the degradation process by decarboxylation.^25,26^ In contrast, the collagen patches exhibited much lower adhesion than the cellulose patch due to the difference in structure between the adhesive polymers used. For TachoSil®, the adhesives are fibrinogen and thrombin, while for Hemopatch®, it is a modified PEG with *N*-hydroxysuccinimide (NHS) groups. Functionalizing this PEG polymer with NHS groups has been shown to allow the correct formation of the adhesive gel upon contact with the proteins in the blood.^27^ Thus, these NHS groups react with the amino groups of the tissue proteins, forming amide-type covalent bonds between the polymer and the tissue. The limitation of this patch type is that this adhesion only occurs when the NHS-PEG forms the hydrogel, so when the tissue is too dry, adhesion is reduced. In addition, as can be seen in the SEM images, the adhesive polymer in Hemopatch® is not homogeneously distributed, and some parts of the patch surface showed no adhesive properties. Some of the problems associated with PEG polymers as an adhesive are related to irregular sealing at the bleeding site or poor fixation with the tissue due to inhomogeneous cross-linking.^28^ In addition, the high hydrophilicity of the PEG polymer may lead to the separation of the hydrogel from the collagen backing, which results in poor hemostasis.

Another aspect is that collagen-based patches presented a significantly slower degradation rate than the oxidized cellulose patch due to collagen’s slower degradation, as reported in the literature.^12^ Collagen degradation can occur by phagocytosis induced by adjacent macrophages and by the secretion of extracellular matrix-degrading enzymes such as metalloproteinases. However, this degradation depends on the type of collagen.^12,29^ Both collagen patches showed less than 60% degradation rates after 25 days of incubation. Compared with Hemopatch®, TachoSil® exhibited a more significant decrease in mechanical properties over time.

Thus, due to the slow degradation rate, super hydrophilicity, high absorption capacity of biological fluids, and moderately good adhesive capacity and hemostasis, Hemopatch® appeared to be the optimal scaffold for incorporating a modified HA hydrogel loaded with chemotherapeutics. In our study, CDDP, a traditional and widely used anticancer agent, and OLA, a novel and promising targeted chemotherapeutic, were selected as model drugs to evaluate and compare the properties of both HAgel and HeHAgel devices.

Hydrogels are widely used as a DDS and have been reported to help prolong drug release in the peritoneal cavity. In the case of CDDP, many researchers have been focused on developing hydrogels for its encapsulation, overcoming the drawbacks of using this drug in intraperitoneal chemotherapy such as the rapid absorption from the peritoneum before being exposed to disseminated nodules. Thus, to avoid a frequent or continuous intraperitoneal administration of the chemotherapeutic agent, one of the main challenges of the hydrogels is to provide a high local concentration of the drug for a longer duration, increasing uptake at the tumor site and, consequently, improve the clinical outcome.^30^ For example, an in situ cross-linkable HA gel was used as a carrier of Pt to develop a targeted and sustained release system.^31^ Although this HA gel reduced the tumor burden more effectively than a free drug solution, the hydrogel alone did not attenuate the CDDP release enough to prevent the initial burst release due to CDDP diffusion capacity through the HA matrix. Furthermore, it is widely described that HA-based hydrogels can be combined with other DDS, such as nanogels^32^ and nanoparticles,^33^ to develop a hybrid system to improve drug release profiles. For example, Ohta and colleagues achieved sustained release over a week by physically encapsulating a CDDP-loaded HA nanogel in an injectable HA hydrogel.^32^

For its part, OLA, like other PARPi, is moving to the forefront of cancer treatment. To the best of our knowledge, the use of hydrogels to carry an OLA has been poorly reported in the literature. Moreover, due to its oral administration, the bioavailability of OLA is relatively low. Therefore, it is a major challenge to effectively deliver OLA and achieve a high drug accumulation in the tumor tissue, for a more precise anticancer drug effect.^20^ By taking advantage of the hydrogels as drug carriers and combining them with another biomaterial that limits drug delivery to specific sites,^34^ we aimed to entrap an HA-based hydrogel loaded with chemotherapeutic drugs (CDDP and OLA) in the polymeric structure Hemopatch® and develop a localized drug delivery device. This way, we maintained a controlled release of CDDP and the resulting release of OLA from the hydrogel matrix for a longer time. Moreover, the Hemopatch® network delayed the diffusion of CDDP from the HA hydrogel, reducing the initial burst release reported for many small drugs. To enhance the sustained release profile further, future studies could incorporate nanoparticle-encapsulated drugs within the hydrogel matrix or multilayered hydrogels to effectively mitigate initial burst release while extending drug release duration.

The metabolic activity assay demonstrated that the HA-based hydrogel and Hemopatch® hemostatic agent were not cytotoxic, confirming their biocompatibility in diverse biomedical applications.^31^ Moreover, this biomaterial has been shown to maintain biocompatibility when combined with Hemopatch®, a widely used safe and effective hemostatic agent and sealant for routine procedures in various surgical disciplines.^12,35−39^ Moreover, loading CDDP and OLA into the HA-based hydrogel and physically encapsulating it into Hemopatch® allowed a significant decrease in cell viability compared with their respective empty carriers, indicating that the chemotherapeutics maintained their function upon release. In addition, although the drug percentage released from the four devices was about 50% at 48 h, which is the same concentration as IC_50_, it resulted in lower cell viability than the free drug. It is widely described that chemotherapeutic drugs used in DDS achieve a more significant cytotoxic effect on cancer cells than when administered freely in a solution.^40−42^ Specifically, HA is a ligand of CD44, which is overexpressed in many tumor cells, so it has emerged as a promising molecule for developing active targeted DDS.^43,44^ For example, a previous report described that encapsulating chemotherapy drugs in an HA gel can increase their uptake by SKOV3 human ovarian cancer cells.^45^ Therefore, an HA-based DDS can achieve a higher effect with the exact dosage.

In conclusion, Hemopatch® was the commercial HP with the most favorable characteristics for its use as a scaffold in controlled-release devices. Besides its sealing and hemostatic properties, this HP showed good wettability, absorption capacity, adhesiveness, and slow degradability, making it the best option for this type of application. From the in vitro studies with an ovarian cancer cell line, we have proved that it is possible to incorporate a hydrogel loaded with CDDP or an OLA that gels in situ in the interior network of the Hemopatch® and allows a sustained release of the chemotherapeutic agents over time, maintaining their function. Furthermore, incorporating HP in the field of DDS could offer other advantages, such as adhesion and hemostatic properties to the resected affected tissue, preventing further growth and metastatic cancer cells. Thus, integrating DDS and HP opens a new window for achieving a localized delivery of the chemotherapeutic drug in peritoneally affected tissue. Future scalability and regulatory approval of a Hemopatch®-based DDS will require careful alignment with manufacturing and regulatory standards. For example, scaling up production must optimize the integration of the hydrogel with Hemopatch® under Good Manufacturing Practices (GMP) to ensure batch-to-batch consistency, sterility, and stability. Moreover, automation of the hydrogel loading and cross-linking processes will be essential. From a regulatory perspective, the system must comply with the regulatory requirements for drug–device combinations including preclinical testing, clinical evaluation, and validation of manufacturing processes.

## 4 Methods

### 4.1 Materials and Reagents

TachoSil® (Baxter International), Hemopatch® (Baxter International Inc.), Veriset^TM^ (Covidien; Mansfield, MA, USA), 24-well Transwell insert (Sarstedt, Numbrecht, Germany), hyaluronic acid (Bioibérica, F002103, Mw 800–1200 kDa, Barcelona, Spain), adipic acid dihydrazide (ADH, Sigma-Aldrich, Madrid, Spain), EDC (Sigma-Aldrich, Madrid, Spain), 1-hydroxybenzotriazole hydrate (HOBt) (Sigma-Aldrich, Madrid, Spain), sodium chloride (NaCl, Vidrafoc, Barcelona, Spain), EtOH (Scharlab, Barcelona, Spain), sodium periodate (NaIO_4_) (Sigma-Aldrich, Madrid, Spain), ethylene glycol (EG) (Sigma-Aldrich, Madrid, Spain), ethyl acetate (Sigma-Aldrich, Madrid, Sapin), acetonitrile (Fisher Chemical, New Hampshire, USA), CDDP (Sellerckchem), OLA (Sellerckchem), Pt 1000 mg/mL standard (Scharlab, Barcelona, Spain), RPMI-1640 medium supplemented with 0.01 mg/mL bovine insulin (Sigma-Aldrich), fetal bovine serum (FBS, Biowest), penicillin/streptomycin (P/S, Biowest), deuterium oxide (D_2_O, Sigma-Aldrich, Madrid, Spain), CellTiter-Glo Luminescent Assay (Promega, Madison, WI, USA), and Presto Blue reagent (Life Technologies, Carlsbad, CA, USA) were used in this study.

### 4.2 Analysis of Hemostatic Patches’ Characteristics and Properties

#### 4.2.1 Microstructure Analysis

The surfaces of the adhesive patches, adhesive and nonadhesive, were imaged on both sides by scanning electron microscopy (SEM) on HitachiS-3400N equipment operating at an accelerating voltage of 2 kV, 4 atm, and 100×.

#### 4.2.2 Contact Angle Measurement

To determine the hydrophobicity of each patch, the water contact angle was measured on both surfaces of different commercial patches. For this purpose, a 10 μL drop of water was placed on the desired patch surface. Subsequently, images of the drops were taken, and the contact angle was measured by using ImageJ software.

#### 4.2.3 Equilibrium Swelling Ratio (ESR) Measurement

Measurements in the ESR were performed to check the water absorption capacity of the different patches. The three different commercial patches (Hemopatch®,TachoSil®, and Veriset^TM^) were incubated at times 0.5, 1, 3, and 24 h in 1 mL of ascitic fluid previously centrifuged at 750*g* for 5 min at 4 °C. Ascitic fluid was obtained from the Institut de Investigació Sanitària de les Illes Balears (IdISBa) Biobank, with the approval of the Ethical Committee (IB 1955–12BIO_ref_21–020) after ethical approval of the project by the CEI-IB (IB 4516/21 PI). The patches were weighed after the corresponding incubation (*W*_s_) and lyophilization (*W*_d_). Finally, the amount of liquid adsorbed was calculated through the weight difference according to the following formula: ESR = (*W*_s_ – *W*_d_)/*W*_d._

#### 4.2.4 Degradation Ratio Measurement

For degradation measurements, patches were incubated in an ascitic fluid. Samples of different patches were cut with a biopsy punch of 6 mm and placed in 24-well plates with 800 μL of ascitic fluid. The ascitic fluid was changed every 2 days, and images were taken at different times. Using ImageJ software, the size reduction of the patches due to degradation was monitored over time by measuring their area at time 0 (area_i_) and at the different time points (area_f_). The degradation percentage was calculated using the following formula: ((area_f_ – area_i_)/area_i_) × 100.

#### 4.2.5 Pull-out Adhesion Measurement

Pull-out adhesion measurements were performed to determine patch variations regarding the tissue attachment strength. This test was carried out in samples of pig liver tissue ex vivo. Pig hepatic lobes purchased from a local butcher shop were cut into pieces according to the following dimensions: 4 cm × 5 cm × 0.5 cm. The cut liver sections were immersed in PBS to remove blotted blood. Patch samples were cut to 1.5 cm × 1.5 cm. After that, patches were adhered to the tissue by wetting the adhesion area with PBS and applying pressure for 1 min. The test was monitored by using a Zwick Z100 tensile speed of 5 mm/min. Tension–elongation curves were registered to determine the maximum force supported by each patch according to its biomaterial formulation. This protocol is an adaptation of the protocol obtained from the literature.^46^

#### 4.2.6 Hemostasis Evaluation In Vivo

##### 4.2.6.1 Animals

Hemostasis was evaluated in vivo in three commercial hybrid pigs. All of them were females, weighing approximately 25–30 kg. The pigs were stabled in enclosures at an average temperature of 22 °C, a relative humidity of 55% ± 10%, and 12 h light/dark cycles. Animals were manipulated according to the ethical committee’s program 2021/23/AEXP guidelines, and care was performed following the standard protocol for animal management. Once the experimental procedure was completed, pigs were sacrificed via intravenous administration of 120 mg/kg of sodic pentobarbital.

##### 4.2.6.2 Preparation and Monitoring

First, the animals were weighed to calculate the dosages of the administered drugs. Intramuscular preanesthesia was administered in the base of the neck using ketamine 10 mg/kg + midazolam 0.5 mg/kg + dexmedetomidine 0.01 mg/kg. Then, hypnosis was performed by continuous propofol administration (1 mg/kg/min) until orotracheal intubation. Multimodal opioid-type analgesia (0.003–0.01 mg/kg/h of fentanyl after an intravenous load of 0.003–0.05 mg/kg), intramuscular nonsteroidal anti-inflammatory before surgery (meloxicam 0.4 mg/kg), and preanesthetic sedative agents for intraoperative analgesia by NMDA antagonist action (ketamine) were further administered. Oro-tracheal-delivered isofuran 1.2–2% or sevoflurane 2.5–3.5% in oxygen/air (50:50) was used as general inhalation anesthesia. In addition, a rebreathing system was used, with lime rolled two l/min during the first 15–20 min and then 0.3–0.5 mL/min. An epidural catheter was placed at the L1 level for locoregional analgesia, and 6 mL of 5% bupivacaine was applied. All animals had atrial marginal vein catheterization and assisted ventilation during the experimental procedure. Vital signs were controlled through heart and respiratory rate, oxygen saturation (SpO_2_), electrocardiogram, temperature, and blood pressure monitoring.

##### 4.2.6.3 Surgery Method

A midline xiphopubic laparotomy was started. The liver was located and mobilized. Three different hepatic lobes were selected for incisions 0.5 cm deep and 1 cm long. An incision was made in three hepatic lobes of each animal, and a different hemostatic state was tested. Lesion’s bleeding was noted once the incision was made, and free bleeding was allowed for less than 5 s. The bleeding in the hepatic periphery was venous blood without arterial jets.

##### 4.2.6.4 Hemostatic Patch Application Technique

The HP was applied following the same protocol for the three commercial brands. The patch was applied directly and centered on the wound. A gauze moistened with a saline solution was used to compress the patch. The time of manual pressure on the gauze was counted for 3 min. After 3 min, the adhesion of the three patches and bleeding were evaluated.

##### 4.2.6.5 Hemostasis Evaluation

Hemostasis was evaluated after 3 min of compression in the wound zone, and then it was classified following the criteria used in the reference article.^47^ The following classification scale was (1) hemostasis with complete adhesion; (2) hemostasis with local delamination of the patch; (3) incomplete hemostasis with adhesive failure; (4) incomplete hemostasis with cohesive failure. We defined adhesive failure as the free outflow of blood from the patch’s perimeter because of patch delamination. Cohesive failure was defined as free blood outflow through the patch.

### 4.3 Synthesis of CDDP and OLA Drug Delivery Devices

#### 4.3.1 Synthesis of the HA Derivative Polymers

Native HA was chemically modified, incorporating amino (−NH_2_) and aldehyde (−CHO) functional groups. Consequently, two different batches, HA-adipic acid dihydrazide (HA-ADH) and HA-aldehyde (HA-CHO), were synthesized following the methodology described previously by Bajaj et al.^48^ Both polymers were purified through dialysis for 7 days, lyophilized, and finally stored at −80 °C under a N_2_ atmosphere until use. The chemical modifications of HA-ADH and HA-CHO were confirmed by proton nuclear magnetic resonance (^1^H NMR) (Bruker Avance 300), using D_2_O as the solvent (Figure 3S).

#### 4.3.2 Synthesis of HA-Based Hydrogels (HAgel)

After synthesizing and characterizing the HA-ADH and HA-CHO polymers, an HA-based hydrogel (40 mg/mL) was further produced. HA-ADH and HA-CHO were dissolved individually in H_2_O Milli-Q. Then, both polymers were mixed in a ratio of 1:1, allowing spontaneous cross-linking and gelation at room temperature. The capability of in situ gelation was confirmed through rheological measurements (Figure 4S).

#### 4.3.3 Synthesis of CDDP-Loaded HA Hydrogels (HAgel-CDDP) and OLA-Loaded HA Hydrogels (HAgel-OLA)

**Figure 5:**
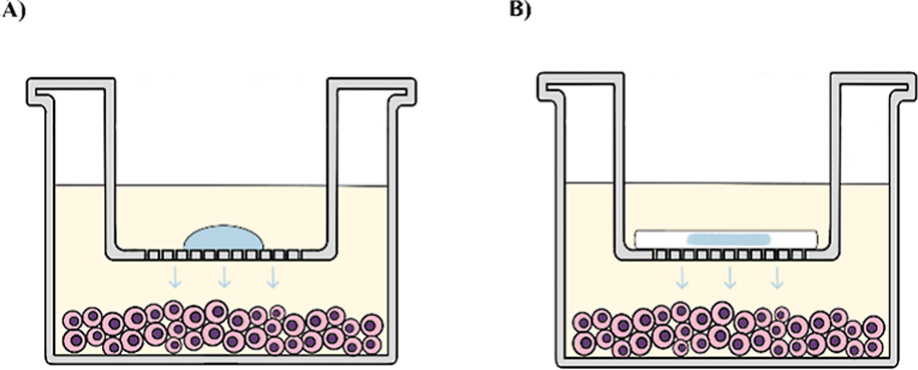
Metabolic activity assay. Schematic drawing synthesizing experimental setup for HAgel-CDDP or HAgel-OLA (A) and HeHAgel-CDDP or HeHAgel-OLA (B) treatment groups. The image shows the OVCAR-3 cell monolayer, the hydrogel (blue), the Hemopatch® (white), the Transwell insert, and the cell culture medium.

After the half-maximal inhibitory concentration (IC_50_) value for CDDP and OLA were determined (Figure 5S), the HA-based hydrogel was loaded with 2× IC_50_ concentration of the respective chemotherapeutics to obtain CDDP-loaded HA-hydrogels (HAgel-CDDP) and OLA-loaded HA-hydrogels (HAgel-OLA). CDDP and OLA solutions were prepared in a H_2_O Milli-Q. HA-ADH and HA-CHO were dissolved in each CDDP or OLA solution and mixed 1:1 to obtain the respective hydrogels. The concentration of each HA polymer was set at 40 mg/mL.

#### 4.3.4 Synthesis of Hemopatch® Loaded with HAgel-CDDP (HeHAgel-CDDP) or HAgel-OLA (HeHAgel-OLA)

Pieces of Hemopatch® were cut with a biopsy punch 6 mm in diameter. Then, 10 μL of HA-ADH and 10 μL of HA-CHO loaded with CDDP or OLA were applied to the patch and immediately absorbed, gelling and cross-linking inside when they mixed, obtaining the HeHAgel-CDDP- or HeHAgel-OLA-loaded device.

To confirm the spontaneous cross-linking and gelation properties of these two HA-modified polymers inside the Hemopatch®, the CDDP release profile in PBS at 1, 6, and 24 h was evaluated and compared with patches loaded with the HA native polymer (HeHAn-CDDP) and with CDDP solution (He-CDDP) (Figure 1S). CDDP released into PBS at these times was determined with ICP–MS. First, the collected samples were evaporated at 70 °C for 24 h. Once dried, 400 μL of HNO_3_ was added and incubated for 24 h at 70 °C to digest all the organic matter. The concentration of CDDP released was determined by measuring platinum (Pt) content using ICP–MS (PerkinElmer Elan DRC-e, Massachusetts, United States). The spectrometer was equipped with a GenTip Crossflow nebulizer, a Ryton Scott Spray Chamber, a 2.0 mm Alumina Injector, a nickel sampler, and skimmer cones. The equipment was controlled by using the ELAN Instrument Control Session (ELAN Version 3.0) program. An autosampler (AS-93 plus) was used to introduce different samples into the ICP–MS. A calibration sample was run with a 1000 mg/L Pt standard. A control was performed without incubation to determine the maximum loading of CDDP in the prepared hydrogels and dissolutions. Residual entrapped CDDP was, in addition, determined.

### 4.4 In Vitro Study of CDDP and OLA Release from HAgel and HeHAgel and Effects on the Metabolic Activity of OVCAR-3 Cells

After evaluation of the release profile at initial time points of HAgel and HeHAgel loaded with CDDP or OLA (Figure 2S), the cell viability and prolonged release rates were also determined. For this purpose, the human epithelial ovarian cancer cell line OVCAR-3 purchased from the American Type Culture Collection (ATCC) (Maryland, USA) was used. Cells were cultured in RPMI-1640 medium supplemented with 0.01 mg/mL bovine insulin, 20% FBS, and 1% P/S and maintained in a humidified 5% CO_2_ incubator at 37 °C. The medium was replaced thrice a week, and cells were trypsinized and subcultured 1:5 every 7 days.

For release studies and metabolic activity experiments, the OVCAR-3 cells were seeded at a density of 3 × 10^4^ cells/well in 24-well plates and cultured with 500 μL of the medium. After 24 h, the medium was removed, cells were cultured with fresh medium, and the respective treatments were applied. Twenty μL of HAgel loaded with the chemotherapeutic drug (HAgel-CDDP or HAgel-OLA) or He pieces containing 20 μL of the respective hydrogel (HeHAgel-CDDP or HeHAgel-OLA) were placed in a 24-well Transwell insert with 0.4 μm pores to avoid direct contact with the cell layer (Figure 5).

After 48 h of treatment, the cultured medium was collected, and the cellular metabolic activity was measured using the Presto Blue reagent following the manufacturer’s protocol. For release studies, Transwell inserts with the respective treatments were placed in new 24-well plates, and the medium was collected and replaced at 72 and 144 h.

CDDP released into the culture media at 48, 72, and 144 h was determined with ICP–MS as previously described. For its part, OLA released at the same time intervals was analyzed using high-performance liquid chromatography (HPLC) (Vanquish Flex, ThermoFisher) with a diode array detector. First, 800 μL of ethyl acetate was added to each 500 μL recollected culture medium. The mixture was briefly vortexed and then centrifuged for 5 min at 16000× g before the supernatant was dried in a N_2_ current and reconstituted with 500 μL of acetonitrile (AcN). A sample volume of 10 μL was injected. The mobile phase consisted of A: water and B: AcN with a flow rate of 0.6 mL/min and a total run time of 20 min. A gradient elution was applied through a reversed-phase Nova-Pak C18 column (4 mm, 150 × 3.9 mm; Waters Corporation, Milford, MA, USA). The following gradient was employed: 0–12 min, 5–95% AcN; 12–15 min, 95% AcN; 15–17 min, 95–5% AcN; 17–20 min, 5% AcN.

Moreover, controls were performed without incubation over time to determine the maximum loading of CDDP and OLA in the prepared hydrogels and solutions. Residual-entrapped CDDP and OLA were also determined using the previously described methods.

## Supporting Information Available

Polymer gelation confirmation inside the Hemopatch®, early release of CDDP and OLA from HA_gel_ and HeHA_gel_ systems, HA-ADH and HA-CHO polymers’ synthesis and characterization, rheological measurements, and CDDP and OLA IC50 determination (PDF)

## Abbreviations

CDDP: cisplatin
CRS: cytoreductive surgery
DDS: drug delivery systems
ERS: equilibrium swelling ratio
HA: hyaluronic acid
HP: hemostatic patches
NHS: *N*-hydroxysuccinimide
OLA: olaparib
PARPi: poly(ADP-ribose) polymerase inhibitors
PC: peritoneal carcinomatosis
PEG: polyethylene glycol
SEM: scanning electronic microscopy
