# Supplementary material for: Evaluation of Different Commercial Sealing Hemostatic Patches for Their Selection as Reservoirs for Localized Intraperitoneal Chemotherapy
Source: ACS Pharmacol Transl Sci. 2025 Jan 8;8(2):499–509. doi: 10.1021/acsptsci.4c00608 (PMC11834274; doi:10.1021/acsptsci.4c00608)
Supplement: Supplementary file 1 — pt4c00608_si_001.pdf [file pt4c00608_si_001.pdf]

Supporting Information

Evaluation of Different Commercial Sealing Hemostatic Patches for Their Selection as Reservoirs for Localized Intraperitoneal Chemotherapy

M. Teresa Perelló-Trias[a,b,c‡], Ana Rodríguez-Fernández[a,b,c‡], Antonio Jose Serrano-Muñoz[a,b,c], Juan J. Segura-Sampedro[a,b,d,e], Pedro Tauler[a,b,c,f], Joana M. Ramis[a,b,c*], Marta Monjo[a,b,c*]

[a] Cell Therapy and Tissue Engineering Group (TERCIT), Research Institute on Health Sciences (IUNICS), University of the Balearic Islands (UIB), 07122 Palma, Mallorca, Spain
[b] Health Research Institute of the Balearic Islands (IdISBa), 07010 Palma, Mallorca, Spain
[c] Department of Fundamental Biology and Health Sciences, University of the Balearic Islands (UIB), 07122 Palma, Mallorca, Spain
[d] General & Digestive Surgery Service, Hospital Universitario la Paz, 28046 Madrid, Spain
[e] Faculty of Medicine, University of the Balearic Islands (UIB), 07122 Palma, Mallorca, Spain
[f] Research Group on Evidence, Lifestyles and Health, Research Institute of Health Sciences (IUNICS), University of the Balearic Islands (UIB), 07122 Palma, Mallorca, Spain

*Corresponding authors. E-mail addresses: joana.ramis@uib.es; marta.monjo@uib.es. Phone: (+34) 971 17 30 00.
‡ These authors contributed equally to this work

1S. Polymer Gelation Confirmation Inside the Hemopatch®

The spontaneous crosslinking capacities of both HA-modified polymers inside the Hemopatch® were confirmed by comparing the CDDP released at short times from parches containing the CDDP solution alone and with HA native polymer loaded with the same CDDP concentration. CDDP solutions were prepared at a concentration of 2xIC_{50} in H_2O MilliQ. HA native, HA-CHO, and HA-ADH were dissolved in this CDDP solution. The concentration of all polymers was set at 40 mg/mL. Pieces of Hemopatch® were cut with a biopsy punch of 6 mm diameter. Then, 20 µL of CDDP solution alone or HA native loaded with this solution were applied to the patch to obtain He-CDDP and $HeHA_n$-CDDP devices, respectively. In the case of $HeHA_{gel}$-CDDP, 10 µL of HA-ADH and 10 µL of HA-CHO loaded with CDDP at the same concentration were applied to the patch. All the samples were placed in 24-well Transwell inserts, and at determined times, the PBS was recollected and replaced with fresh PBS. All data was referred to its respective control group containing the maximum loading of CDDP that could be

incorporated inside the Hemopatch® ($2xIC_{50}$). CDDP released into the PBS at 1, 6, and 24 h was determined with ICP-MS.

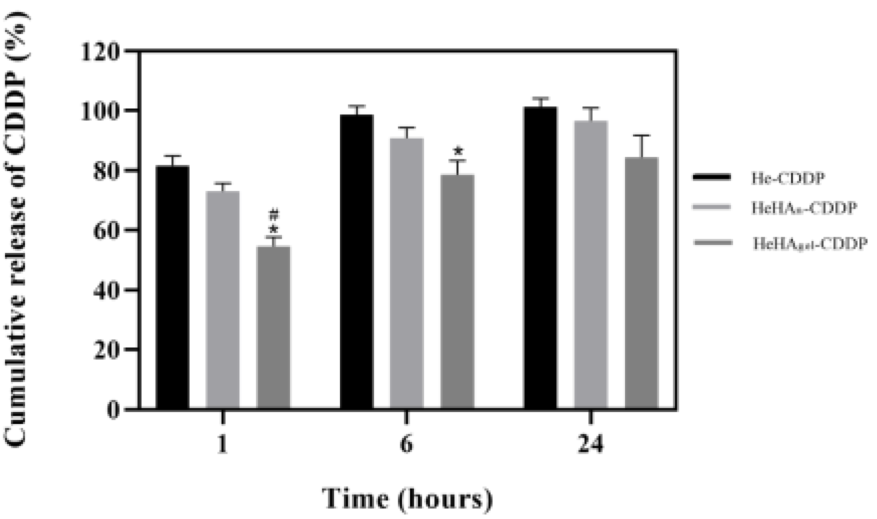

Figure 1S. Amount of CDDP released from He-CDDP, $HeHA_n$-CDDP and $HeHA_{gel}$-CDDP at 1, 6 and 24 h. Platinum content was measured by ICP-MS. Results were statistically compared by the Bonferroni test. Two independent assays were performed (n=6). Values represent the mean ± SEM. $*p < 0.05$ vs. He-CDDP and # $p < 0.05$ vs. $HeHA_n$-CDDP. HA: hyaluronic acid. He: Hemopatch ®. CDDP: cisplatin.

Figure 1S shows that the amount of CDDP released from $HeHA_{gel}$-CDDP is lower than from the HP loaded with the drug in solution alone, even than from HA native polymer, being this difference statistically significant at 1 h. This behaviour indicates that the mix of the two HA-modified polymers created a network that encapsulated the drug.

2S. Early Release of CDDP and OLA from HAgel and HeHAgel Systems

The amount of drug released from the devices at early time points was determined. 20 μL of HA_{gel} loaded with the chemotherapeutic drug (HA_{gel}-CDDP or HA_{gel}-OLA) or He pieces containing 20 μL of the respective hydrogel ($HeHA_{gel}$-CDDP or $HeHA_{gel}$-OLA) were placed in a 24-well Transwell insert with 0.4 μm pores with 500 μL of PBS. At 0.5, 1, 3 and 6 h, the PBS was recollected and replaced with fresh PBS. The amount of CDDP

released was determined using ICP-MS, while the quantity of OLA was determined using HPLC. All data was referred to its respective control group containing the maximum loading of CDDP or OLA.

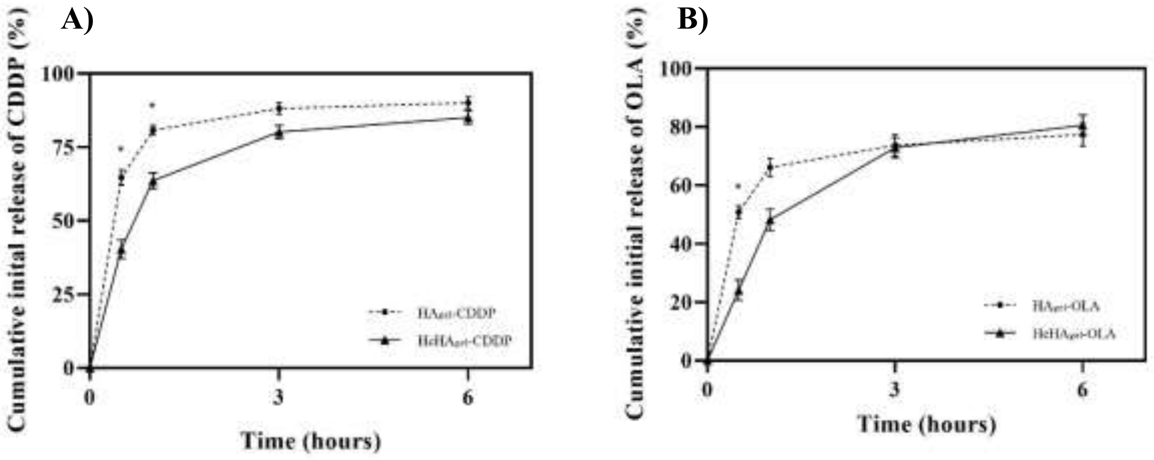

Figure 2S. **(A)** Amount of CDDP released from HA_{gel}-CDDP and $HeHA_{gel}$-CDDP at 0.5, 1, 3 and 6 h. Platinum content was measured by ICP-MS. **(B)** Amount of OLA released from HA_{gel}-OLA and $HeHA_{gel}$-OLA at 0.5, 1, 3 and 6 h. OLA content was measured by HPLC with a diode array detector. Results were statistically compared by the Bonferroni test. Three independent assays were performed (n=9). Values represent the mean ± SEM. *$p < 0.05$ vs. HA_{gel}-CDDP (A) or HA_{gel}-OLA (B). HA: hyaluronic acid. He: Hemopatch®. CDDP: cisplatin. OLA: olaparib.

3S. HA-ADH and HA-CHO Polymers' Synthesis and Characterization

3.1S. Synthesis of derivative HA-amino (HA-ADH) and HA-aldehyde (HA-CHO)

HA chains were modified with the aim to obtain HA-amino derivatives (ADH) and HA-aldehyde derivatives (HA-CHO) polymers following the protocol described previously by Bajaj *et al.*[48]

To synthesize the HA-ADH derivative polymer, 500 mg (1.32 mmol; Mw = 1.2x106 g/mol) of HA was dissolved in H_2O MilliQ at 3 mg/mL at room temperature. HA was reacted with a 30-fold molar excess of ADH (6887.9 mg; 39.54 mmol) at pH 6.8. Then, 1012.2 mg (5.28 mmol) of EDC / 735.5 mg (5.28 mmol) HOBt dissolved in 5 mL of DMSO/H2O 1:1 was added to the solution. The reaction was carried out overnight in the dark at pH 6.8 and room temperature. Then, the pH was adjusted to 7.0, and the product was dialyzed at 37 ºC for one week. Next, 5 % NaCl was added, and the modified HA was precipitated using EtOH. Finally, the precipitate was recovered and re-dissolved in H_2O miliQ at a concentration of 5 mg/mL.

HA-CHO derivative polymer was produced by reacting 1000 mg of HA (2.64 mmol; Mw = 1.2x106 g/mol) dissolved in H_2O MilliQ at 3 mg/mL with equimolar $NaIO_4$. The reaction was carried out for 2 h at room temperature in the dark. Then, 671 µL of EG was added to inactive the unreacted periodate.

HA-ADH and HA-CHO products were lyophilized for one week and stored at -70ºC under a N_2 atmosphere until use.

3.2S. ^{1}H-NMR Characterization of HA, HA-ADH and HA-CHO to Confirm the Modification of HA

From ^{1}H-NMR, the peaks show the characteristic signals of the functional groups existing in HA before and after chemical modification (**Figure 2S**). HA is a polymer with a very high molecular weight (Mw=1.2 x 106 g/mol). Hence, a broad multiplet appears between approximately d =3.0-3.9 ppm, corresponding to the signal of the protons appearing in the rings. These signals cannot be assigned to a specific proton due to the overlap of the peaks. From the ^{1}H-NMR of HA-ADH, the signal at d =1.89 ppm highlighted in green

(letter a) corresponds to the N-acetyl methyl functional group. The signals at d =1.53 ppm, d =2.14 ppm and d =2.27 ppm, highlighted in blue, red, and purple, respectively, correspond to the methylene groups of ADH and confirm the successful chemical modification of HA by ADH.

In the ^{1}H-NMR of HA-CHO, the green colour shows only the characteristic signal of the N-acetyl methyl functional group at d =1.91 ppm. The difference between HA before and after oxidation shows only some changes in the broad multiplet region between approximately d =3.0-3.9 ppm, which corresponds to the signal of the sugar ring protons. Therefore, ^{1}H-NMR did not clearly reveal the presence of aldehyde groups due to partial modification of the polymer. However, the small changes in the proton region of the sugar rings allow confirming a new chemical structure in HA.

In addition, as confirmation of polymer modification, we can use the variation in viscosity of the reaction mixture, which visually decreased during the reaction time, indicating a slight loss of molecular weight due to oxidation.

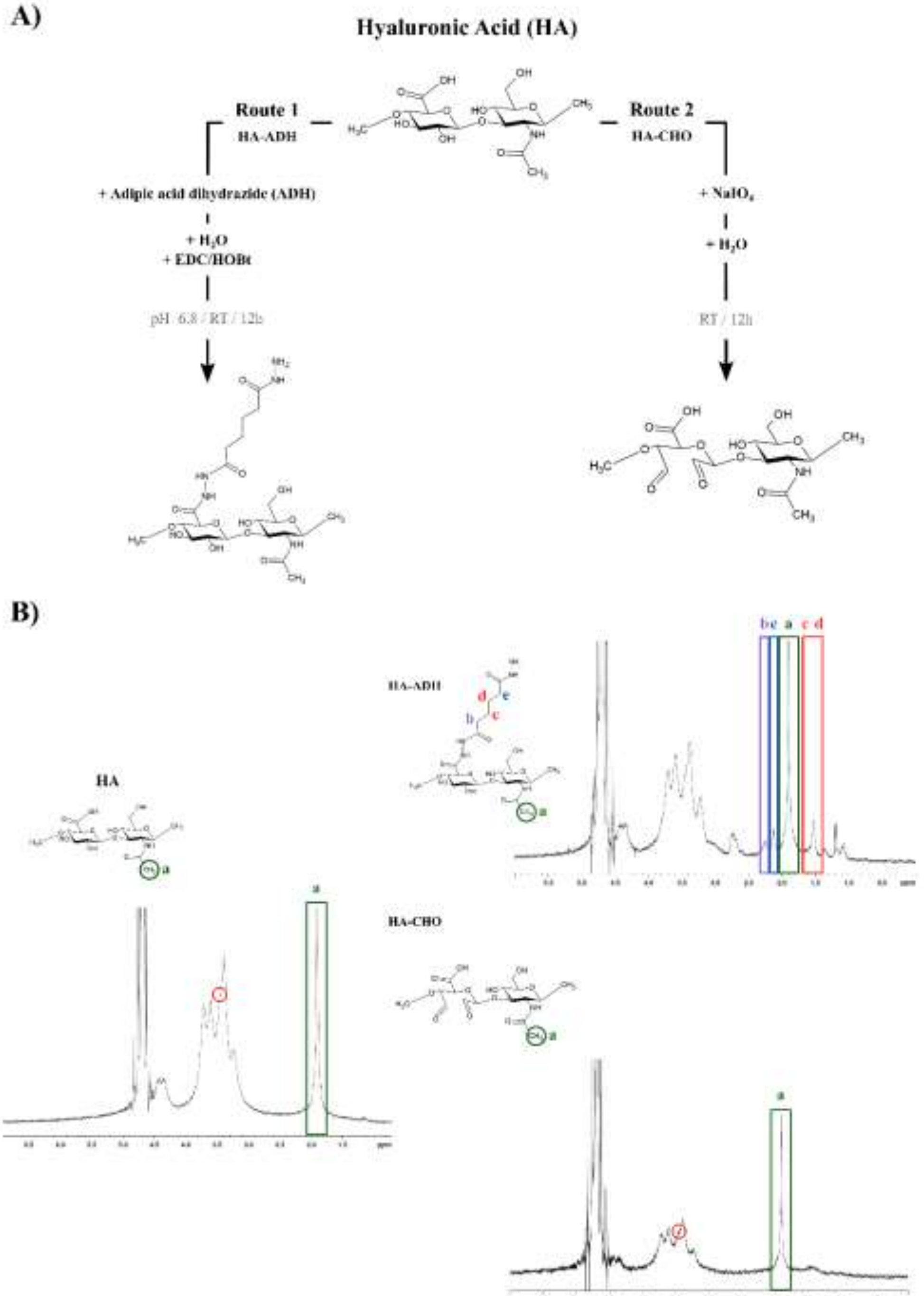

Figure 3S. HA-ADH and HA-CHO derivative polymers schematic synthesis (**A**) and characterization (**B**). Red (c+d): signal at d=1.53 ppm was assigned to the methylene group. Green (a): signal at d=1.89 ppm was assigned to N-acetyl methyl group. Blue (e): signal at d= 2.14 ppm was assigned to methylene group. Purple (b): signal at d=d.27 ppm was assigned to methylene group.

4S. Rheological Measurements

Rheological measurements of the HA_{gel} were performed using an MCR 302e modular compact rheometer (Anton Paar, Germany). The sample was measured on a 25 mm plate diameter. The working gap was 0,4 mm, and the measurements were conducted at 25ºC and a frequency of 1 Hz. Storage modulus (G′) and loss modulus (G″) within the linear viscoelastic range (LVR) were determined. The assays were carried out five times (n=5).

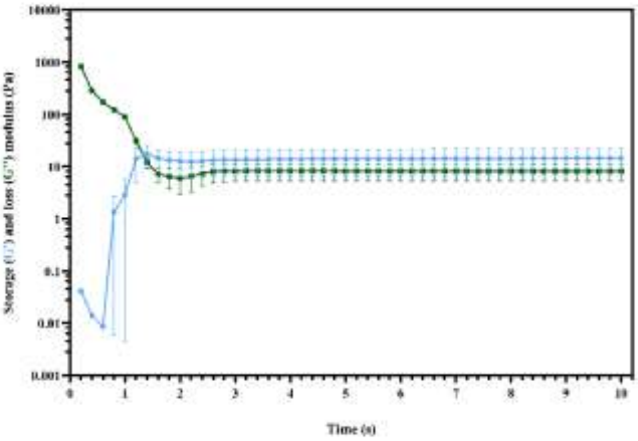

Figure 4S. Gelation kinetics of HA_{gel} measured by rheology. The storage modulus (G') and loss modulus (G") were recorded as a function of gelation time. Gel point is determined as the crossover point of G' and G". Values represent the mean ± SEM.

5S. CDDP AND OLA IC_{50} Determination

For half-maximal inhibitory concentration (IC_{50}) determination of CDDP and OLA, the number of viable cells was determined by quantification of cellular ATP levels, which signals the presence of metabolically active cells, using the CellTiter-Glo Luminescent Assay. OVCAR-3 cells were seeded in 96-well plates at a density of $5x10^3$ cells/well and cultured as usual in their respective supplemented medium. After overnight incubation, the medium was aspirated and replaced with fresh medium containing different concentrations of CDDP (ranging from 0.01-100 µM) or OLA (ranging from 0.01- 400

µM). After 48 h of treatment, cell viability was measured according to the manufacturer's instructions. The luminescence signal was detected through a Synergy Mix Scanning Spectrophotometer (Biotek). The dose-response curves and the IC_{50} were calculated using non-linear regression with GraphPad Prism X software (GraphPad Software, CA, USA).

The IC_{50} value for the OVCAR-3 cells was 4.61 µM for CDDP and 226.3 µM for OLA, indicating that these cells are more responsive to CDDP than OLA. For the upcoming studies, the HA-based hydrogels were loaded with twice the CDDP or OLA concentration required to inhibit cell growth by 50% (2xIC_{50}).

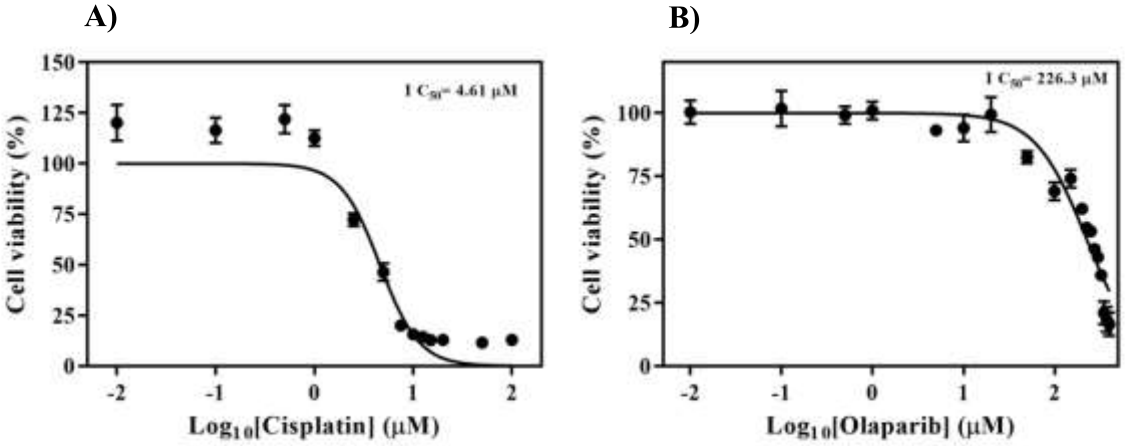

Figure 5S. Dose-response curve and half-maximal inhibitory concentration (IC_{50}) value of OVCAR-3 after treatment with CDDP (**A**) or OLA (**B**). Cell viability was assessed at 48 h post-treatment by measuring the cellular ATP levels using a luminescent CellTiter-Glo Assay. The curve is the result of 3 independent assays. Values represent the mean ± SEM. IC_{50} was calculated from the dose-response curve using non-linear regression with GraphPad Prism.
